# Post-Colectomy Cacosmia: The Role of Anxiety and Sensory Distortion in Postoperative Recovery

**DOI:** 10.1192/j.eurpsy.2025.1026

**Published:** 2025-08-26

**Authors:** Y. Mashayekhi, M. Marudkar, R. Sherrat

**Affiliations:** 1 Mental health liaison service, Leicester Royal infirmary, Leicester, United Kingdom

## Abstract

**Introduction:**

Cacosmia, the perception of neutral odors as foul, is an unusual and distressing postoperative complication. This case focuses on a 66-year-old female who experienced cacosmia after a colectomy, raising questions about the role of anxiety in postoperative sensory distortions. While colectomy is commonly associated with physiological complications, emerging research indicates that psychological factors, particularly anxiety, may influence sensory processing. Addressing these interactions is crucial for improving outcomes in patients with psychiatric comorbidities undergoing surgery.

**Objectives:**

1. To examine the influence of pre-existing anxiety on the development of sensory distortions, specifically cacosmia, following colectomy.
2. To investigate the relationship between the gut-brain axis and psychological factors, including anxiety, in the context of postoperative recovery.
3. To highlight the gaps in the literature regarding sensory distortions in surgical patients and to propose potential areas for future research.

**Methods:**

**Patient:** A 66-year-old female with a history of anxiety disorder developed cacosmia two weeks after undergoing colectomy.

**Symptoms:**

She reported that food smelled like feces despite normal postoperative recovery and the absence of physical abnormalities.

**Literature Review:**

**a** search was conducted using databases such as PubMed and Scopus with terms like *cacosmia*, *postoperative sensory distortion*, *anxiety and surgery*, and *gut-brain axis.* Articles exploring the relationship between anxiety and sensory distortions in surgical patients were included.

**Results:**

**Gut-Brain Axis:** The gut-brain axis is essential in regulating emotions and sensory processing. Disruptions from surgery can affect neurotransmitters, contributing to both anxiety and sensory misinterpretations like cacosmia. **Anxiety’s Role:** Anxiety is known to heighten sensory perception. The patient’s pre-existing anxiety likely amplified her awareness of sensory stimuli, leading to distorted odor perceptions.

**Literature Gap:**

While the role of anxiety in surgical recovery is recognized, there is a lack of research specifically addressing sensory distortions like cacosmia in the postoperative period.

**Conclusions:**

This case underscores the importance of considering psychological factors, especially anxiety, in postoperative sensory distortions like cacosmia. The interaction between the gut-brain axis and sensory perception in anxious patients highlights the need for a more comprehensive, multidisciplinary approach to postoperative care. Current gaps in the literature suggest a need for future research on how anxiety and sensory processing intersect after surgery. Investigating preoperative interventions such as cognitive-behavioral therapy (CBT) or pharmacotherapy may help mitigate sensory distortions and improve postoperative recovery outcomes in patients with anxiety.

**Disclosure of Interest:**

None Declared

